# Evolution and synergy of healthcare security policies for rare diseases in China: a quantitative content analysis

**DOI:** 10.3389/fpubh.2026.1864284

**Published:** 2026-07-21

**Authors:** Jingying Gao, Chaojie Liu, Xinyang Wang, Haojia Cai, Yuqing Tang

**Affiliations:** 1 School of Medicine and Health Management, Tongji Medical College, Huazhong University of Science and Technology, Wuhan, Hubei, China; 2 School of Psychology and Public Health, La Trobe University, Melbourne, VIC, Australia; 3 Major Disciplinary Platform under Double First-Class Initiative for Liberal Arts at Huazhong University of Science and Technology (Research Center for High-Quality Development of Hospitals), Wuhan, Hubei, China; 4 Key Research Institute of Humanities and Social Sciences of Hubei Provincial Department of Education, Wuhan, Hubei, China

**Keywords:** rare diseases, healthcare security, policy synergy, health policy, China

## Abstract

**Background:**

Managing rare diseases presents substantial challenges to achieving universal health coverage. Despite growing policy interest, limited research has explored the evolution and synergy of healthcare security policies targeting rare diseases. This study addresses this gap through a case study of China.

**Methods:**

An innovative analytical framework was developed, structured around three dimensions of policy synergy: horizontal, vertical, and temporal. A comprehensive dataset of national and provincial policy documents related to healthcare security for rare diseases was systematically collected. Analytical techniques included quantitative content analysis, word frequency analysis, and semantic network analysis.

**Results:**

A total of 314 policies enacted between 2001 and 2025 remained effective at the time of data collection, showing a general upward trend in both quantity and policy strength. However, the majority of policies were notices and announcements, which typically carry lower enforcement authority than ordinances and regulations. While a diverse array of policy instruments was employed, horizontal synergy across sectors remained limited. Vertically, national policies emphasized screening and support for children with rare diseases, whereas provincial policies focused more on outpatient service subsidies.

**Conclusion:**

Rare diseases have gained increasing attention within China’s healthcare security policy landscape. Nonetheless, significant gaps in horizontal and vertical policy synergy persist. A systems-based approach is recommended to enhance policy integration and effectiveness.

## 1 Introduction

Rare diseases have increasingly been recognized as a significant public health challenge, profoundly affecting patients, their families and caregivers, healthcare systems, and society at large (1). Although each rare disease affects fewer than 1 in 2,000 individuals, the cumulative global burden is substantial, with over 7,000 identified rare diseases and an estimated prevalence of 3.5 to 5.9% in the general population—equating to approximately 263–446 million people worldwide (2, 3).

Healthcare services for individuals with rare diseases are often under-prioritized, particularly in low- and middle-income countries (2). The economic burden on affected individuals and families is considerable, with costs per patient per year estimated to be 10 times higher than those for common diseases (4, 5). The costs are also overwhelmingly indirect/non-health in nature (6). Due to high treatment costs and limited market demand, health insurance programs frequently struggle to negotiate affordable access to therapies, necessitating state intervention (7, 8).

High-income countries have adopted various mechanisms to address these challenges (9). In Australia, ultra-orphan drugs (10) are funded through the Pharmaceutical Benefits Scheme (PBS) under the Rule of Rescue (RoR) or via the Life Saving Drugs Program (LSDP), which applies more stringent and objective criteria (11, 12). In the United States, the Inflation Reduction Act (IRA) of 2022 empowered Medicare to negotiate drug prices, although orphan-only drugs were excluded to preserve innovation incentives (13, 14). In contrast, Canada lacks a national orphan drug strategy, resulting in lower approval and reimbursement rates for orphan medicines compared to the United States and EU (15).

In Central and Eastern Europe, policy frameworks for rare diseases remain underdeveloped. While some countries offer exemptions from health technology assessments (HTA) for certain rare disease treatments, others lack dedicated reimbursement pathways (8, 16). Examples include Bulgaria’s automatic reimbursement for listed rare disease treatments, Romania’s coverage of 70 orphan medicinal products, and Russia’s federal and regional reimbursement schemes (17, 18).

In low- and middle-income countries, attention to rare diseases remains limited. Initiatives such as Brazil’s Casa Hunter and Peru’s Fondo Intangible Solidario de Salud (FISSAL) provide financial support to low-income patients, while Colombia’s legislation recognizes rare disease patients as a protected class (19).

Rare diseases and orphan drugs place considerable strain on healthcare systems (20). Fragmented policies and siloed decision-making contribute to inefficiencies, resource duplication, and inequities in care (21). Poor coordination exacerbates fragmentation, undermines accountability, and impedes effective policy implementation (22). Addressing these challenges requires integrated, multi-sectoral approaches, particularly in contexts like China, where economic disparities and uneven resource allocation complicate policy responses (23).

There is growing consensus on the need for enhanced policy synergy to improve effectiveness and sustainability (24). According to the Organization for Economic Co-operation and Development (OECD), policy synergy encompasses three dimensions: horizontal, vertical, and temporal (25). Horizontal synergy promotes coherence across sectors and minimizes conflicting objectives. Vertical synergy ensures alignment between policy intentions and service delivery across government levels. Temporal synergy supports long-term policy coherence by anticipating future developments and costs. In intergovernmental contexts, horizontal synergy refers to coordination among departments at the same level, while vertical synergy involves hierarchical relationships between agencies (26).

Policy synergy analysis has been applied in various domains, including illicit drug policy (22), science and technology (27), energy (28), and environmental governance (29, 30). However, research on the coordination of healthcare security policies for rare diseases remains limited. In China, healthcare security policies for rare diseases are jointly formulated and implemented by central and provincial governments, involving key agencies such as the Healthcare Security Administration and the National Health Commission. In this study, “healthcare security” is operationally defined as a multi-level financing system, encompassing basic medical insurance, catastrophic disease insurance, medical assistance programs, commercial health insurance, and charitable contributions.

This study addresses this gap by conducting a case study in China, examining the horizontal, vertical, and temporal synergies of healthcare security policies for rare diseases. China released its first rare disease catalog in 2018 and updated it in 2023, listing 207 conditions (31). Yet, only around 90 related products are included in the National Reimbursement Drug List (NRDL), with regional governments authorized to expand coverage (32, 33).

Previous research has highlighted progress in diagnosis, treatment, research and development, and drug accessibility for rare diseases in China (34–36), while also noting regional disparities and coordination challenges (36). Fragmented decision-making, overlapping responsibilities, and institutional conflicts threaten the effectiveness of policy initiatives. This study aims to systematically analyze the evolution and synergy of healthcare security policies for rare diseases in China, contributing to both academic literature and practical policy formulation.

## 2 Methods

### 2.1 Data collection

This study systematically collected policy documents issued by central and provincial governments in China concerning healthcare security for rare diseases. The data collection was restricted to documents published prior to April 25, 2025, the designated retrieval date. A keyword-based search strategy was employed, incorporating terms related to rare diseases (e.g., chronic diseases, special diseases, specific disease types, serious and catastrophic diseases), outpatient care, special/specific medicines, and medical insurance (e.g., basic medical insurance, catastrophic disease insurance, medical assistance, assistance projects, medical reimbursement, and medicines catalogs).

To ensure comprehensiveness, two separate searches were conducted:

1) Peking University LAW database: This database is recognized as the most professional and comprehensive legal and policy repository in China, containing over 4.6 million documents related to central and regional legislation (37).
2) Governmental websites: A total of 163 official websites were searched, including those of general government bodies and departments responsible for healthcare security, health, human resources and social security, and civil affairs. Supplementary material provides a complete list of the websites included.

Policy documents were screened for eligibility based on the following criteria:

1) Issued by central or provincial governments. Documents from municipal or lower levels were excluded due to their high convergence with provincial policies in terms of content and structure.
2) Explicit reference to healthcare security for rare diseases.
3) Designed to promote the healthcare security for rare diseases.
4) Were valid and publicly available at the time of data collection.

### 2.2 Data analysis

The quantitative analysis was operationalized through keyword frequency analysis, co-occurrence network analysis, and policy document quantification. These analytical procedures constitute the operational implementation of the content analysis framework in this study. This analytical approach is informed by established policy text quantification studies (38–40).

Policy synergy analysis is fundamentally grounded in the examination of policy texts (30, 38, 41), typically employing content analysis and social network analysis to assess coherence and coordination. Synergies are often quantified across multiple dimensions of policy goals or sub-goals. Common approaches to policy text analysis include the use of specialized text analysis tools (42), policy modeling consistency index models (37, 43), and policy quantification tables (44). These methods frequently rely on expert evaluations, which, while insightful, introduce a degree of subjectivity into the analysis (40).

#### 2.2.1 Policy synergy analysis framework

**Figure 1:**
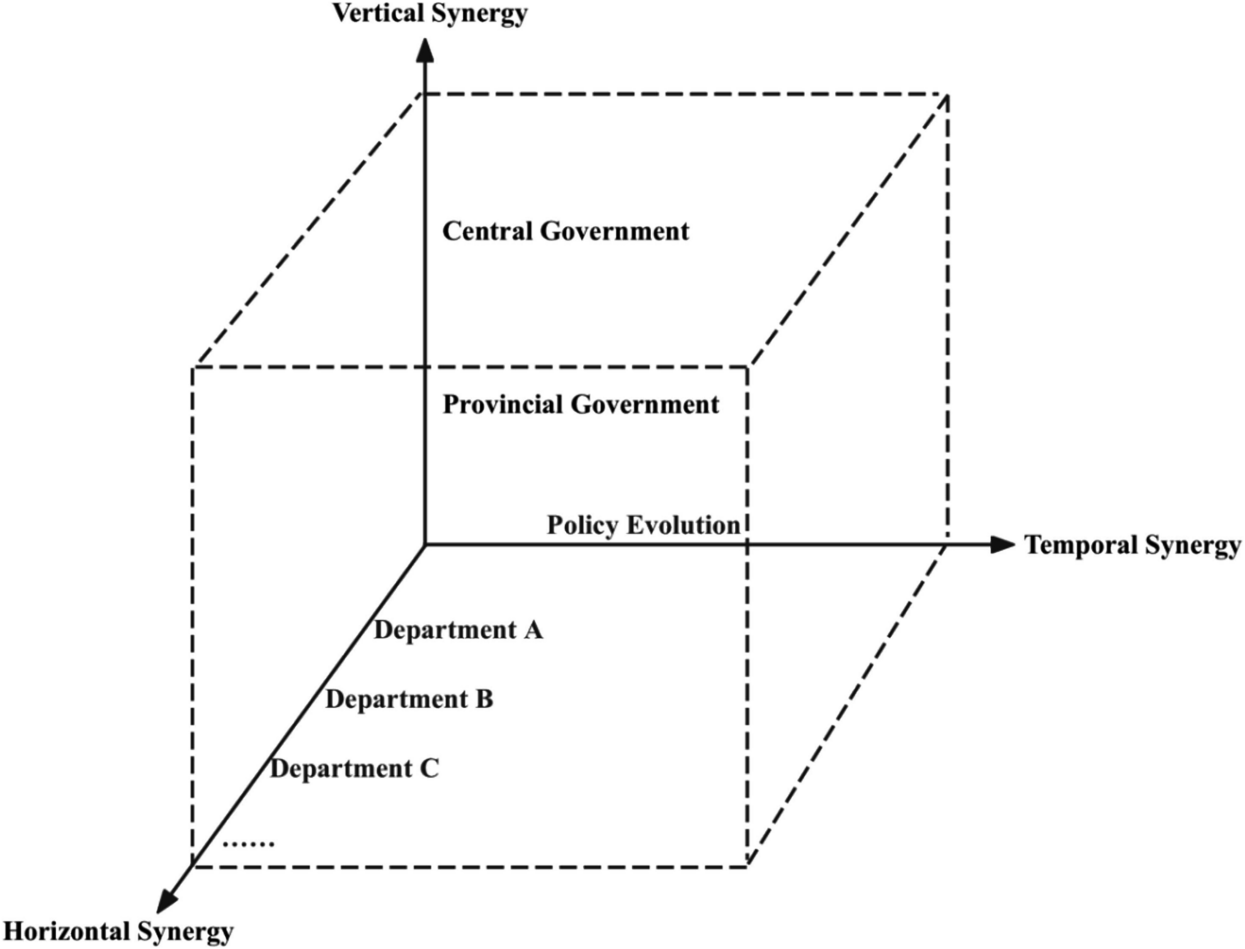
Policy synergy analysis framework.

This study employed an analytical framework (Figure 1) grounded in the three dimensions of policy synergy: horizontal, vertical, and temporal (25, 38). This multidimensional approach enables a systematic evaluation of the coherence and coordination of healthcare security policies for rare diseases across administrative levels, sectors, and time.

#### 2.2.2 Horizontal synergy

In this study, horizontal policy synergy refers to the composition and degree of coordination among policies issued by different departments within the same level of government. Horizontal synergy emphasizes collaborative relationships across governmental agencies. To account for structural changes over time, issuing authorities were grouped according to the current administrative framework, with historical agency alignments considered to ensure consistency. For the analysis of issuing agencies, each agency was counted based on its occurrence in policy documents. In jointly issued documents, each participating agency was counted once. Therefore, proportions were calculated based on the total frequency of agency occurrences rather than the number of policy documents.

The use of joint issuance as a proxy for inter-agency coordination has been widely adopted in quantitative policy analysis, as it reflects formal collaboration intensity among governmental departments (39). To quantify the synergy effect among policy makers, the policy-level horizontal synergy score was calculated as the number of issuing departments minus one. The annual horizontal synergy was then computed using Equation 1:

$$C_{i}=\sum_{j}(N_{\mathrm{ij}}-1)$$ (1)

where *C_i_* represents the overall horizontal synergy of healthcare security policies for rare diseases in year *i*; *N_ij_* denotes the number of issuing departments for policy *j* in year *i*.

#### 2.2.3 Vertical synergy

Vertical synergy in this study refers to the coordination between central and provincial government policies, with a particular focus on policy content synergy. To assess this, the study employed word frequency analysis and semantic network analysis to minimize subjectivity, both widely used techniques in bibliometric and social network research.

Word frequency analysis quantifies the occurrence of specific keywords within policy texts, with higher frequencies indicating greater relevance to the policy’s core themes and future directions (45). This study utilized ROST CM6, a text mining software developed for Chinese-language content analysis (46). The process involved two steps: (1) Word segmentation of central and provincial policy documents using the software’s built-in parser. (2) Frequency analysis of segmented texts to generate keyword statistics.

To further explore content relationships, semantic network analysis was conducted. A semantic network consists of nodes (representing entities, concepts, or themes) and edges (indicating co-occurrence within sentences) (47). The degree centrality of a node reflects its prominence within the network—larger nodes indicate more central themes with broader connectivity.

High-frequency keywords were extracted to construct a co-occurrence matrix, which was then visualized using NetDraw (48), a specialized tool for social network analysis. The resulting co-occurrence network diagram of policy keywords illustrates the thematic structure and interconnections within central and provincial policy texts, enabling a comparative assessment of vertical policy synergy.

#### 2.2.4 Temporal synergy

This study regards temporal synergy as the continuity and stability of policies at different stages. To assess the temporal evolution of healthcare security policies for rare diseases in China, this study evaluated policy strength based on two key factors: the issuing authority and the legal effect of the policy document. Different types of policy documents carry varying degrees of normative and binding force, and the hierarchical level of the issuing organization also influences the authority and impact of the policy.

Following the approach of Chen et al. (40), policy strength was quantified by assigning scores that reflect both the type of document and the level of the issuing body (Table 1). This approach is consistent with prior policy quantification studies, where higher-level authorities and more formalized legal documents are generally associated with stronger binding force and greater implementation impact. It should be noted that the ordinal scoring scheme (1–4) does not assume perfectly linear or equal intervals between categories. Instead, it provides a simplified proxy for relative policy strength, a common practice in policy text quantification.

**Table 1:** Policy strength of different types of policy documents.

Policy strength	Policy type
4	Ordinances and regulations issued by the state council
3	Notices issued by the central government and regulations from various ministries
2	Notices from various ministries and regulations from multiple provinces
1	Notices from provincial-level units

## 3 Results

Between 2001 and 2025, a total of 314 relevant policy documents remained in effect as of the date of data collection. Of these, 21 were issued by the central government, while 293 originated from provincial governments. In terms of policy strength, three documents received a score of 4, five scored 3, 37 scored 2, and 269 scored 1.

Rare diseases were addressed through a variety of mechanisms, including basic medical insurance, medical assistance, catastrophic disease insurance (a form of supplementary coverage), commercial health insurance, and charitable aid. These efforts involved multiple government agencies. The Healthcare Security Administration is primarily responsible for developing and implementing reimbursement and payment standards for rare diseases under medical insurance. The Health Commission oversees the diagnosis, treatment, and clinical management of rare diseases. The Civil Affairs Bureau manages medical assistance programs for rare diseases.

China does not have dedicated mandatory pricing or reimbursement policies specifically for rare diseases. However, based on the status of local healthcare funds and financial resources, several provinces have introduced policies to ensure access to medications for specific rare conditions. The scope of coverage and reimbursement levels vary significantly across regions.

### 3.1 Policy temporal evolution analysis

**Figure 2:**
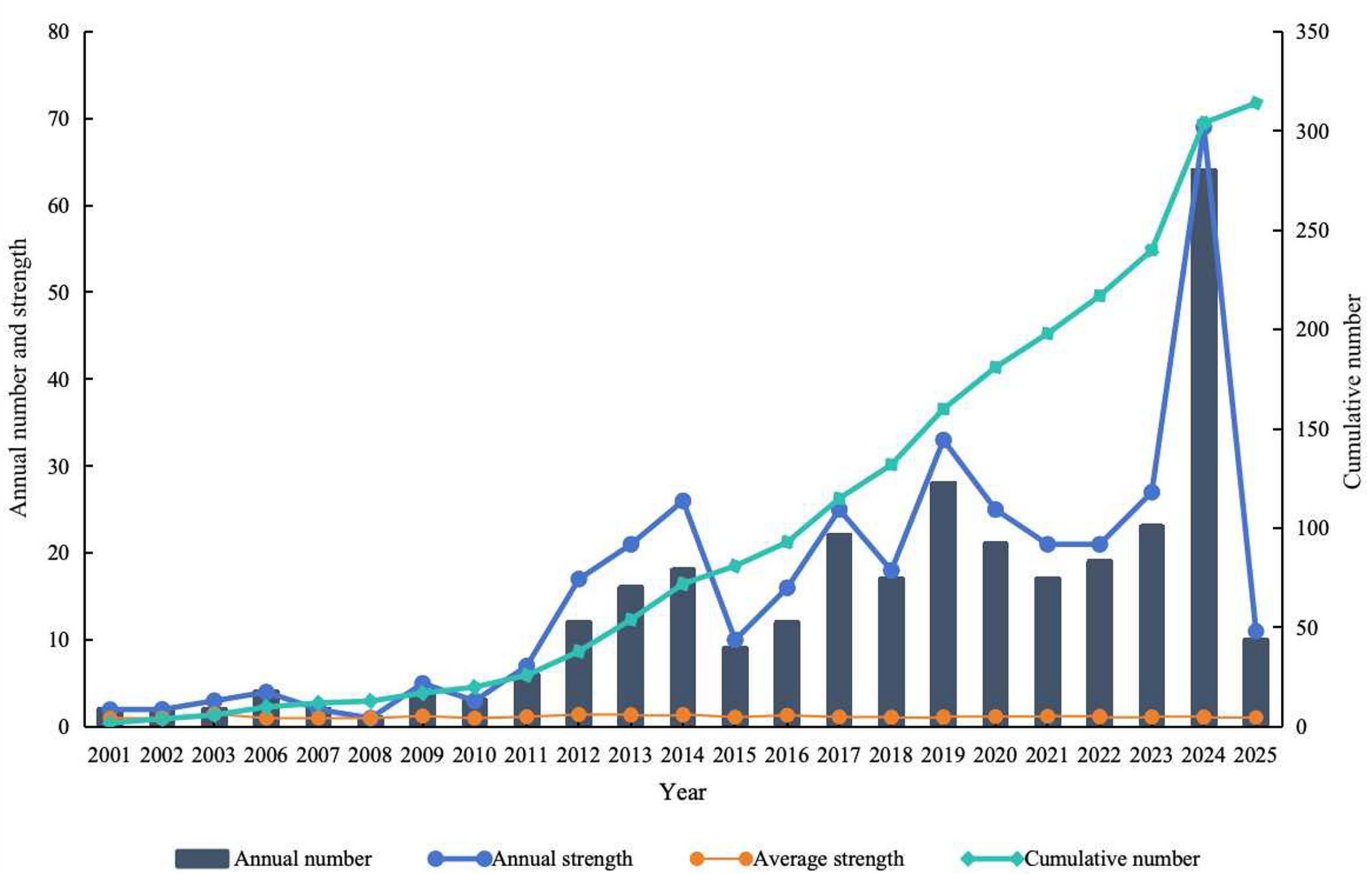
Number and strength of newly published policies by year.

Since 2010, the number and strength of newly published policies have exhibited a generally upward yet fluctuating trend. Prior to 2011, policy activity was minimal, with both the quantity and strength of policies remaining very low. Between 2011 and 2024, there was a marked increase in both the number and strength of newly published policies, reaching a peak in 2024. Although the overall trajectory indicates growth in both dimensions, the average strength of newly published policies has shown a declining trend over time (Figure 2).

### 3.2 Horizontal synergy analysis

#### 3.2.1 Composition of policy makers

**Figure 3:**
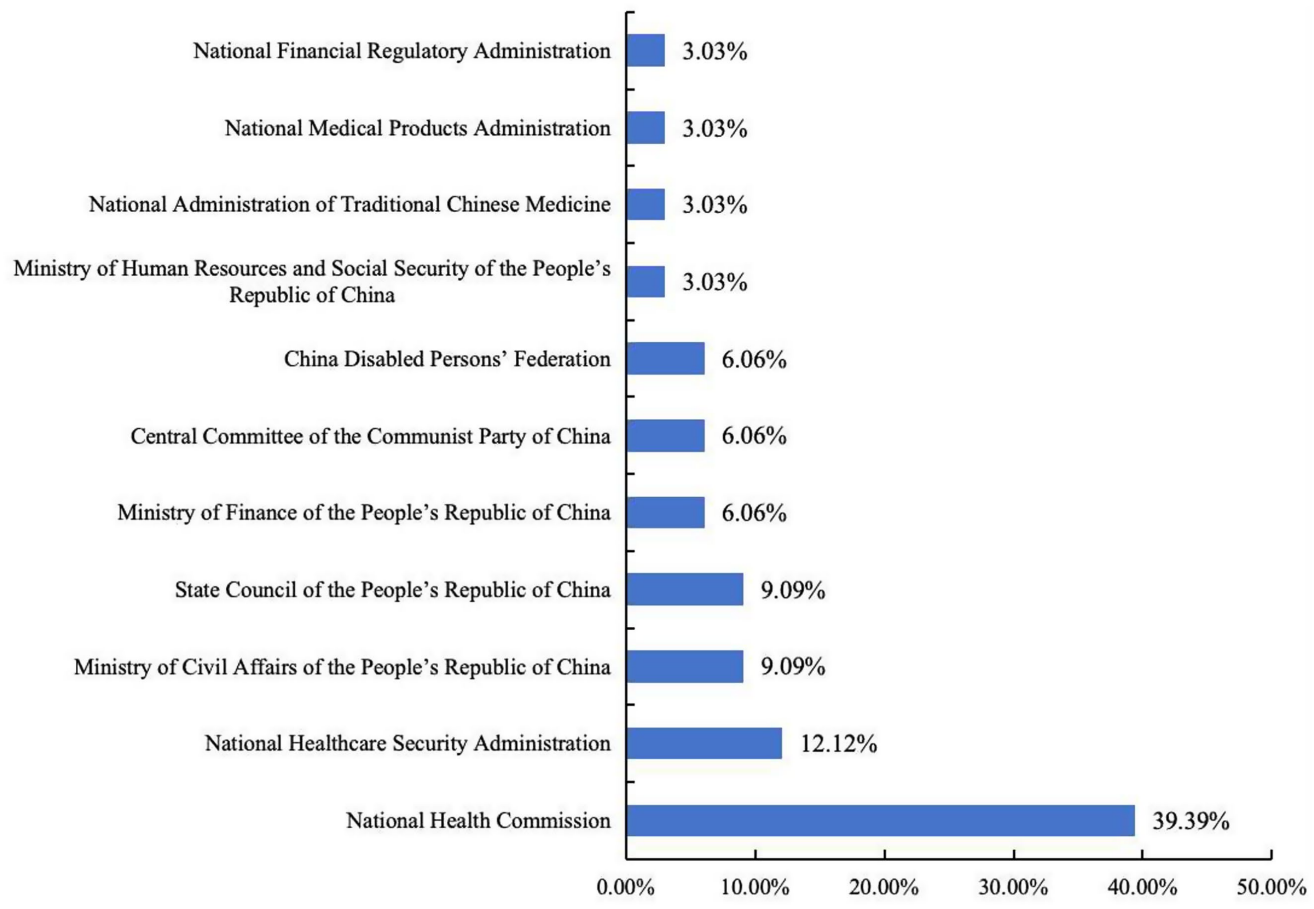
Proportion of policy documents issued (including joint issuance) by central agencies.

**Figure 4:**
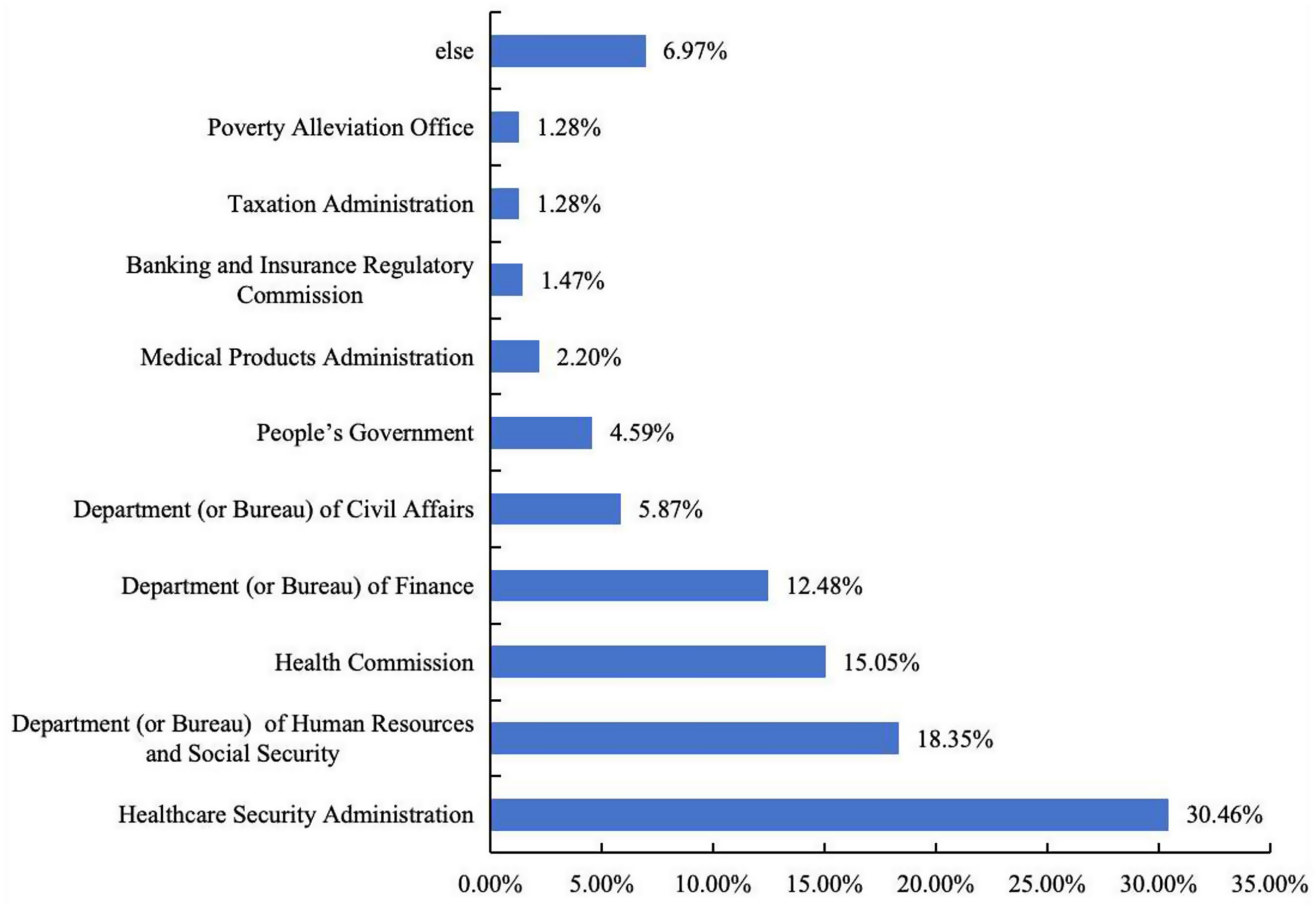
Proportion of policy documents issued (including joint issuance) by agencies in the provincial governments.

The effective policies were issued by 11 central agencies and 29 provincial agencies. At the central level, the most frequently cited agency was the National Health Commission (*n* = 13), followed by the National Healthcare Security Administration (*n* = 4) (Figure 3). At the provincial level, the Healthcare Security Administration and the Department (or Bureau) of Human Resources and Social Security were the most frequently involved issuing agencies, accounting for 30.46% and 18.35% of all agency issuances (Figure 4).

Approximately 15% of central policy documents involved the State Council or the Central Committee of the Communist Party, compared to a much lower proportion of provincial policy documents that referenced Provincial People’s Governments.

#### 3.2.2 Synergistic analysis of policy makers

**Figure 5:**
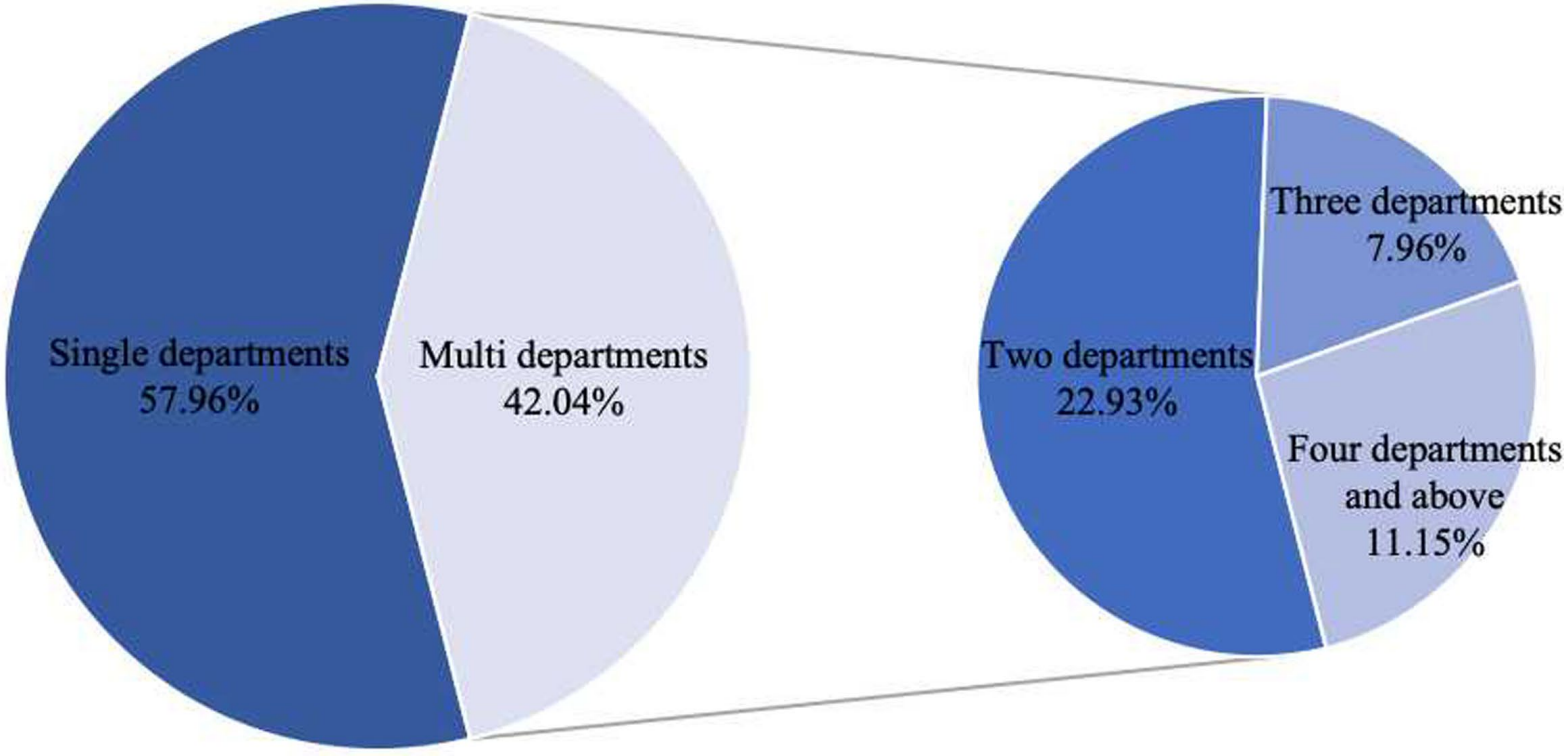
Joint issuance of health security policy documents for rare diseases.

**Figure 6:**
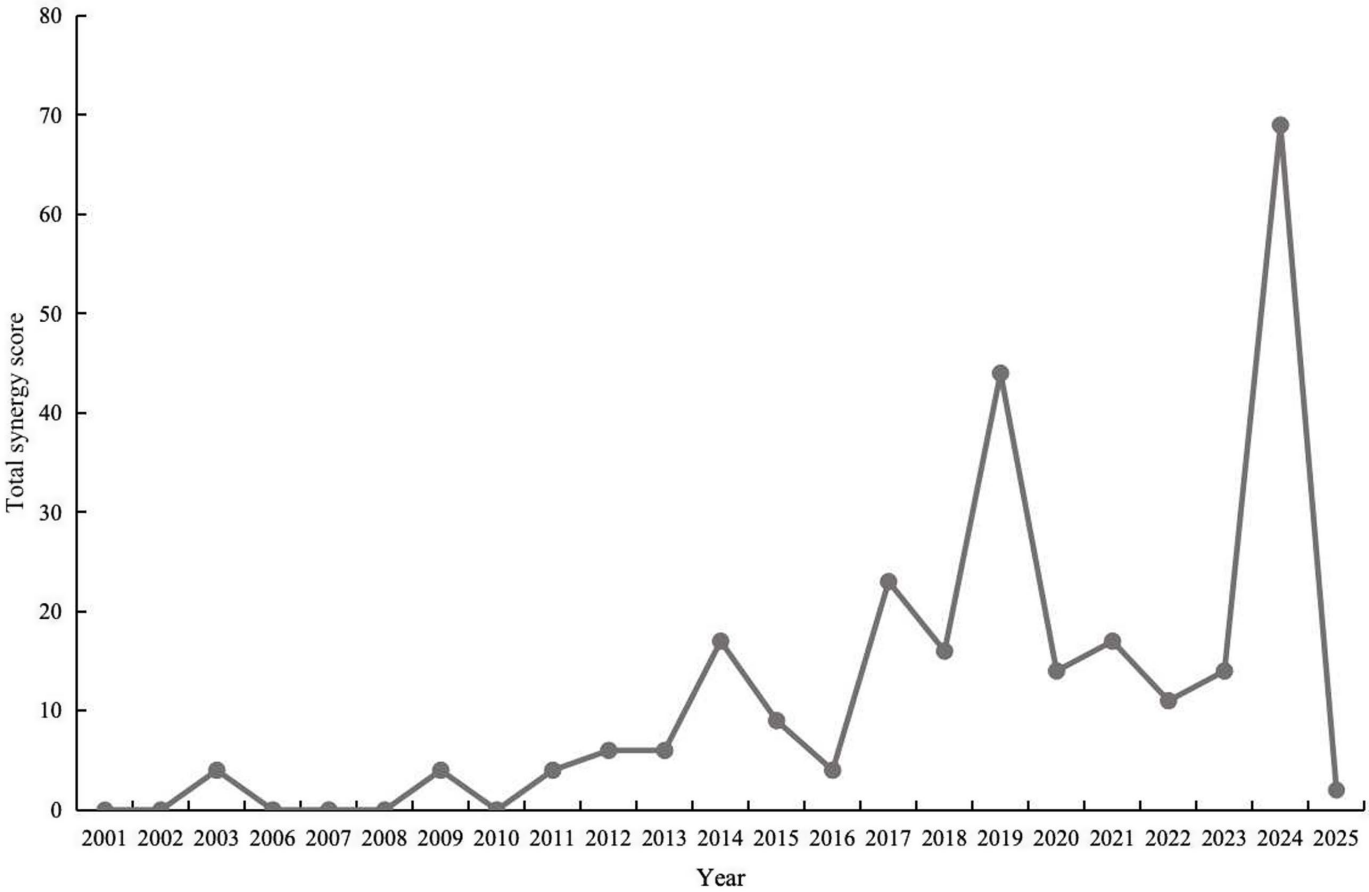
Horizontal synergy scores of health security policy documents for rare diseases by year.

About 42.04% of all documents relating to healthcare security policies for rare diseases in China were jointly issued by multiple departments (Figure 5). Among these, policies jointly issued by two departments were the most common, representing 22.93%. Rather than increasing steadily over time, inter-ministerial collaboration in rare disease healthcare security policy followed a fluctuating upward trend, with two distinct peaks (Figure 6). The first peak occurred in 2019, likely influenced by the establishment of the National Healthcare Security Administration in 2018. That year, five departments—including the National Health Commission—jointly released the “First Batch of Rare Disease Catalog.” The second peak was observed in 2024, possibly linked to the joint formulation and release of the “Second Batch of Rare Disease Catalog” in 2023 by the National Health Commission and six other departments, covering a total of 86 rare diseases. Additionally, the impact of COVID-19 disruptions was evident in the timeline.

### 3.3 Vertical synergy analysis

**Figure 7:**
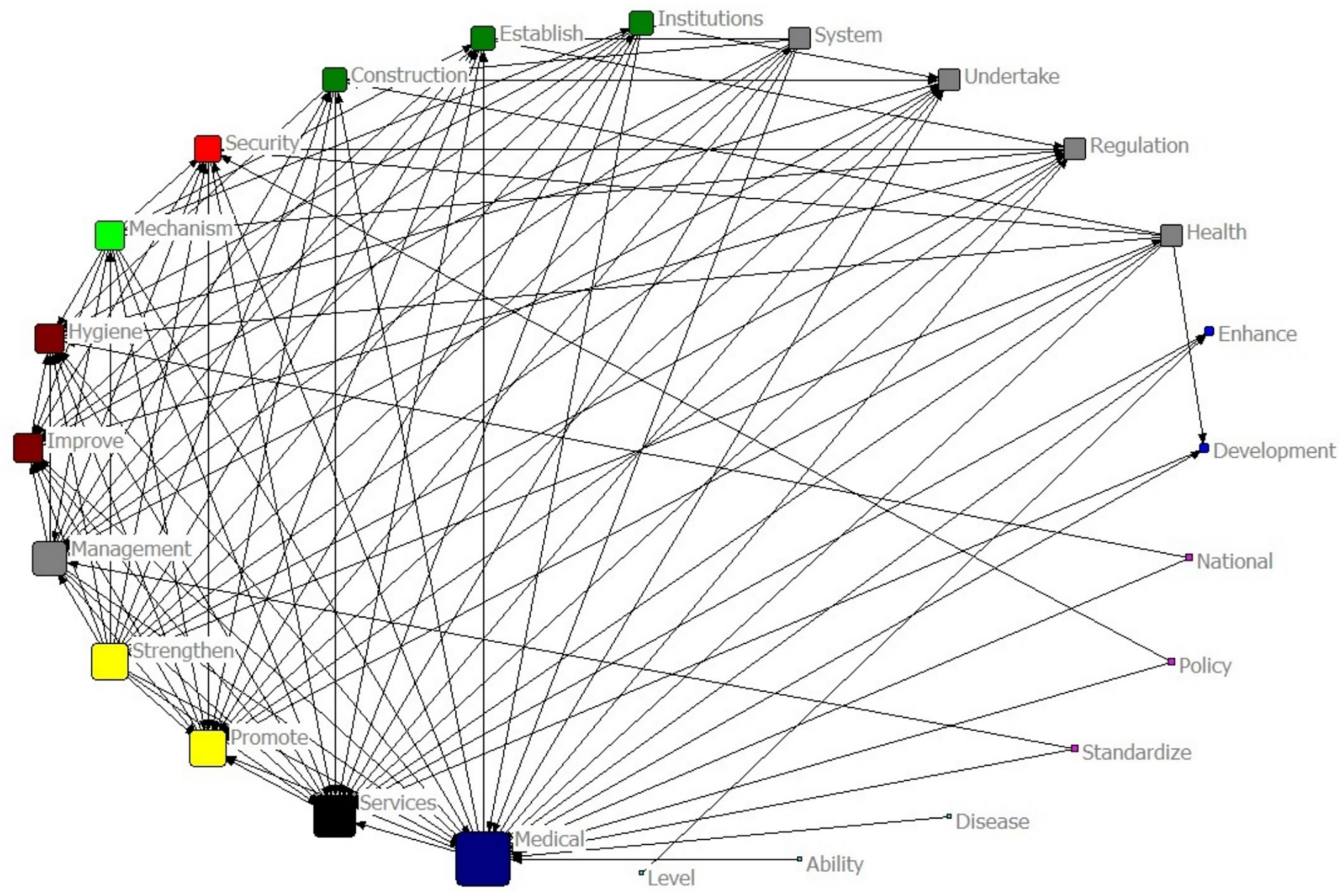
Co-occurrence network diagram of policy keywords at the central level.

**Figure 8:**
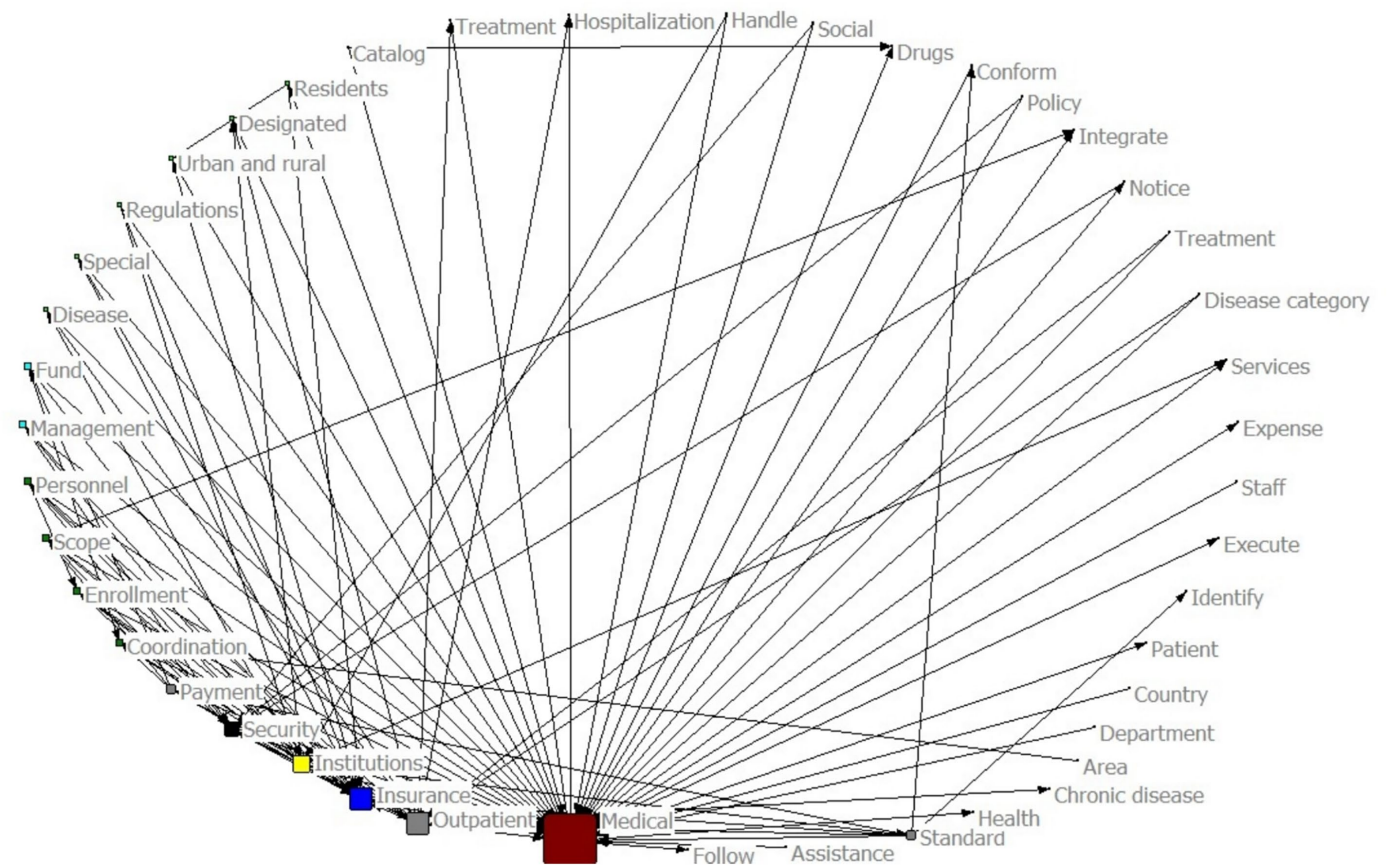
Co-occurrence network diagram of policy keywords at the provincial level.

Both central and provincial policies emphasized the importance of medical coverage for rare diseases. At the central level, key terms frequently emphasized in policy texts included *services*, *program*, *mechanism*, *regulation*, and *child*. In contrast, provincial-level policies highlighted terms such as *outpatient*, *payment*, *standard*, *designated*, and *fund* (Table 2). A distinctive feature of provincial policies was the emphasis on *outpatient*, *standard*, *treatment*, *payment*, and *designated*. Additionally, keywords with high centrality in central-level policies included *medical*, *services*, *promotion*, *strengthen*, and *management* (Figure 7), while provincial-level policies show high centrality for *medical*, *outpatient*, *insurance*, *institutions*, and *security* (Figure 8).

**Table 2:** Frequency of key words in healthcare security policies for rare diseases at the central and provincial levels.

Central policies				Provincial policies			
Medical	737	Drugs	221	Medical	10,830	Management	2,398
Hygiene	544	Mechanism	216	Insurance	5,323	Fund	2,340
Health	481	Regulation	216	Institutions	4,853	Personnel	2,262
Services	446	Establish	204	Outpatient	4,549	Enrollment	2,218
Security	391	Child	199	Security	4,193	Coordination	2,154
Management	361	National	195	Standard	3,848	Hospitalization	1973
Strengthen	348	Disease	186	Treatment	3,595	Conform	1963
Insurance	335	System	178	Drugs	3,270	Scope	1933
Institutions	329	Assistance	172	Payment	3,157	Residents	1928
Construction	289	Enhance	170	Disease	2,885	Special	1903
Promote	287	Social	160	Designated	2,666	Diagnosis	1886
Undertake	267	Policy	160	Examination	2634	Regulations	1844
Program	229	Hospitals	150	Disease category	2,605	Benefits	1760
Improve	226	Reform	146	Assistance	2,457	Patient	1,648
Development	223	Major	143	Hospitals	2,433	Identify	1,646

## 4 Discussion

Our research identified a general upward trend in both the number and strength of policies related to healthcare security for rare diseases in China. However, most of these policies are notices and announcements, which are considered low in effectiveness. High-impact documents such as ordinances and regulations remain scarce. While a wide range of policy makers are involved, horizontal policy coordination across departments needs further improvement. In terms of vertical coordination, central-level policies tend to focus on assistance for children, whereas provincial-level policies are more oriented toward outpatient coverage. Central policies emphasize humanitarian aspects, while provincial policies focus more on medication and treatment.

The temporal evolution of policy issuance reflects the government’s sustained interest in healthcare security for rare diseases. Notably, there was a rapid increase in both the number and strength of policies between 2011 and 2024. This surge may be attributed to the central government’s 2012 initiative to accelerate healthcare security for catastrophic diseases among rural residents, prompting various departments and provincial governments to introduce relevant policies. The release of the first and second batches of the Rare Disease Catalog in 2018 and 2023, respectively, led to two significant peaks in policy activity. These catalogs had a substantial impact on policy development, consistent with findings by Li et al. (36), who noted that rare disease policy trends in China are closely tied to the release of these catalogs. Additionally, the issuance of the *Opinions on the Establishment of the Healthcare Security Treatment List System* by the National Healthcare Security Administration further influenced regional policy improvements.

Despite the overall upward trend, the average strength of policies has declined. The predominance of low-effectiveness documents such as notices and announcements has diluted the overall policy strength. This suggests that the perceived increase in policy strength is driven more by quantity than by legal authority. A robust policy system requires a greater proportion of high-quality, high-effectiveness documents. International experience from countries such as the United States, Japan, and the European Union demonstrates that legislation is a critical step in improving conditions for rare disease patients (49). In China, Jiangsu Province passed the *Jiangsu Provincial Healthcare Security Regulations* in January 2023, which came into effect in June 2023. Article 31 of the regulation establishes a mechanism for guaranteeing medication for rare diseases, providing legal protection for the province’s healthcare security system.

China’s healthcare security policies for rare diseases are diverse and involve a broad array of policy makers. However, a few departments are responsible for most policy formulation. Cross-departmental collaboration is evident, with nearly half of the policies jointly issued by multiple departments. Jointly issued policies tend to be more influential, as they reflect interdepartmental cooperation (37). Moving forward, it is essential to develop sustainable pricing and reimbursement models and enhance global collaboration across public and private sectors (50). China has increasingly recognized the importance of synergistic policy implementation, as reflected in the growing number of joint documents. The average degree of synergy shows two peaks. After 2024, synergy levels declined, possibly indicating a transition from an exploratory phase to a period of steady, comprehensive policy refinement. Nonetheless, the pattern of inter-ministerial cooperation remains relatively fixed, and further efforts are needed to enhance policy coordination and operational effectiveness.

In terms of policy themes, central-level policies emphasize assistance for children, likely reflecting a focus on prevention and early intervention. Provincial-level policies, on the other hand, prioritize outpatient coverage, addressing the long-term treatment needs of patients. This divergence results in inconsistencies in implementation across regions, potentially affecting policy uniformity and accessibility. It reflects both complementary functions, with the central level prioritizing early intervention and provinces addressing long-term care, and potential fragmentation, as inconsistent provincial standards may undermine policy uniformity and equitable access. Clearly, national reimbursement policies for rare disease patients need to be more standardized (51). Current policies are largely localized, with varying standards across provinces, which may lead to inequities in patient treatment. Strengthening top-level policy design at the central level is essential to promote a more unified healthcare security system for rare diseases.

Health insurance plays a vital role in improving access to orphan drugs (52). However, reimbursement processes and policies vary significantly across provinces in China, complicating access for patients who need treatment outside their home region. Although some provinces now allow cross-regional reimbursement, the complexity and lack of transparency in insurance policies remain major barriers for rare disease patients (53). Studies show that many rare disease drugs are not included in the medical insurance catalog, resulting in significant disparities in reimbursement rates among patients under the same insurance type (54). Expanding the scope of healthcare security and including more rare disease drugs in the insurance catalog is necessary to promote equity. Differences in the number of reimbursed drugs may also be influenced by national medicine budgets (55–58).

Variations in national legislation, healthcare budgets, insurance systems, and reimbursement policies contribute to global disparities in access to orphan drugs. Reimbursement policies for expensive orphan drugs tend to be stricter, and regional differences are significant (59). Countries have adopted various approaches to address rare disease challenges, but there is no universal solution. Policies must be tailored to each country’s specific context and needs (60). For example, Singapore’s Ministry of Health and the SingHealth Fund jointly established the Rare Disease Fund, a charitable initiative combining community donations and government matching contributions to support citizens with rare diseases requiring high-cost treatments (61). Internationally, countries such as Australia, United Kingdom, and Republic of Korea have implemented distinct risk-sharing agreements, which Singapore’s framework does not currently emulate (9).

These horizontal and vertical synergy gaps may hinder patient access to diagnosis and care in practice. For instance, limited inter-departmental coordination could complicate reimbursement procedures, while inconsistent central-provincial priorities may create regional disparities in treatment continuity and outpatient coverage. This underscores the need for stronger policy synergy to translate policy intentions into improved patient outcomes. These regional variations also raise concerns about equity and financial protection, as patients in different provinces face unequal reimbursement standards and out-of-pocket burdens, challenging the goal of universal health coverage.

This study suffered from several limitations. First, other policy domains, such as diagnosis, screening, registry systems, and research, were beyond the scope of this study. Future research could extend the analysis to these areas for a more comprehensive perspective. Second, this study is mainly based on policy text analysis, reflecting policy intentions rather than actual implementation effects. Policy documents cannot reveal practical obstacles such as informal coordination at the implementation level. Future research can delve into the actual mechanisms and gaps in policy implementation through semi-structured interviews (with policymakers, hospital administrators, patient advocates) or case studies. Third, the temporal analysis of this study only includes current effective policies and does not include policies that have expired or been abolished. This bias may lead to underestimation of early policy activities. Future research can incorporate all historical policies (including those that have already expired) to more accurately depict the trajectory of policy evolution. Fourth, future research could also draw on patient-level data, hospital records, or survey-based measures to assess whether these policies have translated into improved access to services and enhanced financial protection for rare disease patients.

## 5 Conclusion

This study reveals the evolutionary trends, inter-agency synergies, and regional disparities in China’s healthcare security policies for rare diseases. While there has been a general upward trend in both the number and strength of policies, most documents are notices and announcements with limited legal effectiveness, and high-impact regulatory instruments remain scarce. Although policy makers are broadly engaged, horizontal coordination across departments requires further improvement. In terms of vertical synergy, central-level policies tend to focus on assistance for children, whereas provincial-level policies are more inclined toward outpatient coverage.

The findings of this study not only serve as a valuable reference for policymakers in China but also offer insights for other low- and middle-income countries. Future research could expand data sources and conduct cross-country comparative analyses to generate broader lessons and inform the optimization of global health insurance systems for rare diseases.

## References

[1] Delaye J Cacciatore P Kole A. Valuing the “burden” and impact of rare diseases: a scoping review. Front Pharmacol. (2022) 13:914338. doi: 10.3389/fphar.2022.914338, 35754469 PMC9213803

[2] The Lancet Global Health. The landscape for rare diseases in 2024. Lancet Glob Health. (2024) 12:e341. doi: 10.1016/S2214-109X(24)00056-1 38365397

[3] Nguengang Wakap S Lambert DM Olry A Rodwell C Gueydan C Lanneau V . Estimating cumulative point prevalence of rare diseases: analysis of the orphanet database. Eur J Hum Genet. (2020) 28:165–73. doi: 10.1038/s41431-019-0508-0, 31527858 PMC6974615

[4] Angelis A Tordrup D Kanavos P. Socio-economic burden of rare diseases: a systematic review of cost of illness evidence. Health Policy. (2015) 119:964–79. doi: 10.1016/j.healthpol.2014.12.016, 25661982

[5] Chiesi Global Rare Diseases. (2022). The burden of rare diseases: an economic evaluation. Available online at: https://www.ultragenyx.com/wp-content/uploads/2024/05/Chiesi-EconomicBurdenofRareDiseasesFeb.-2022.pdf (Accessed March 26, 2025)

[6] Ng QX Ong C Yaow CYL Chan HW Thumboo J Wang Y . Cost-of-illness studies of inherited retinal diseases: a systematic review. Orphanet J Rare Dis. (2024) 19:93. doi: 10.1186/s13023-024-03099-9, 38424595 PMC10905859

[7] Malinowski KP Kawalec P Trąbka W Czech M Petrova G Manova M . Reimbursement legislations and decision making for orphan drugs in central and eastern European countries. Front Pharmacol. (2019) 10:487. doi: 10.3389/fphar.2019.00487, 31139080 PMC6518361

[8] Nicod E Whittal A Drummond M Facey K. Are supplemental appraisal/reimbursement processes needed for rare disease treatments? An international comparison of country approaches. Orphanet J Rare Dis. (2020) 15:189. doi: 10.1186/s13023-020-01462-0, 32690107 PMC7370450

[9] Ng QX Ong C Chan KE Ong TSK Lim IJX Tang ASP . Comparative policy analysis of national rare disease funding policies in Australia, Singapore, South Korea, the United Kingdom and the United States: a scoping review. Heal Econ Rev. (2024) 14:42. doi: 10.1186/s13561-024-00519-1, 38896399 PMC11186122

[10] Clifford KA Levine AA Enright DE Neumann PJ Chambers JD. The health benefits, costs, and cost-effectiveness of ultraorphan drugs. Value Health. (2024) 27:1656–61. doi: 10.1016/j.jval.2024.07.005, 39094687

[11] Lee CK Proudfoot EM O'Leary BA. Access to medicines for rare diseases in Australia: the current climate for reimbursement. Value Health. (2017) 20:A567–8. doi: 10.1016/j.jval.2017.08.959

[12] Priest VL Dummett H. PHP10 Australia’s rule of rescue and the life saving drugs programme: same-same but different? Value Health. (2012) 15:A14–5. doi: 10.1016/j.jval.2012.03.087

[13] Hwang TJ Kesselheim AS Rome BN. New reforms to prescription drug pricing in the US: opportunities and challenges. JAMA. (2022) 328:1041–2. doi: 10.1001/jama.2022.15268, 35984652

[14] Vogel M Zhao O Feldman WB Chandra A Kesselheim AS Rome BN. Cost of exempting sole orphan drugs from Medicare negotiation. JAMA Intern Med. (2024) 184:63–9. doi: 10.1001/jamainternmed.2023.6293, 38010643 PMC10682941

[15] Rawson NSB Adams J. Orphan drugs approved in Canada: health technology assessment, price negotiation, and government formulary listing. Expert Opin Orphan Drugs. (2024) 12:1–11. doi: 10.1080/21678707.2024.2313766

[16] Skóra K Augustyńska J Leszczynska A Seweryn M. HPR48 reimbursement policies regarding rare diseases in central and eastern European countries. Value Health. (2022) 25:S241. doi: 10.1016/j.jval.2022.09.1180

[17] Young KE Soussi I Toumi M. The perverse impact of external reference pricing (ERP): a comparison of orphan drugs affordability in 12 European countries. A call for policy change. J Mark Access Health Policy. (2017) 5:1369817. doi: 10.1080/20016689.2017.1369817, 29081920 PMC5645904

[18] Pejcic AV Iskrov G Jakovljevic MM Stefanov R. Access to orphan drugs—comparison across Balkan countries. Health Policy. (2018) 122:583–9. doi: 10.1016/j.healthpol.2018.04.009, 29729905

[19] Taruscio D Salvatore M Lumaka A Carta C Cellai LL Ferrari G . Undiagnosed diseases: needs and opportunities in 20 countries participating in the undiagnosed diseases network international. Front Public Health. (2023) 11:1079601. doi: 10.3389/fpubh.2023.1079601, 36935719 PMC10017550

[20] Hanchard MS. Debates over orphan drug pricing: a meta-narrative literature review. Orphanet J Rare Dis. (2025) 20:107. doi: 10.1186/s13023-025-03634-2, 40055799 PMC11887186

[21] Christensen T Lægreid P. The whole-of-government approach to public sector reform. Public Adm Rev. (2007) 67:1059–66. doi: 10.1111/j.1540-6210.2007.00797.x

[22] Hughes CE Ritter A Mabbitt N. Drug policy coordination: identifying and assessing dimensions of coordination. Int J Drug Policy. (2013) 24:244–50. doi: 10.1016/j.drugpo.2012.08.004, 23036652

[23] Chen J Li Y Chang J. Rare diseases in developing countries: insights from China’s collaborative network. Int J Health Plann Manag. (2024) 39:135–40. doi: 10.1002/hpm.3712, 37776316

[24] Zhang H Zhu L Zeng C Chen X. Text mining and quantitative research of medical service policy: Sichuan province as an example. Front Public Health. (2020) 8:509842. doi: 10.3389/fpubh.2020.509842 33490004 PMC7820774

[25] OECD (2000) Government coherence: the role of the centre of government. Available online at: https://one.oecd.org/document/puma/mpm(2000)3/en/pdf (Accessed March 30, 2025).

[26] Sun T Wen X. The regional environmental governance from the perspective of the intergovernmental relations: a quantitative analysis of the air pollution prevent policy texts in the Beijing-Tianjin-Hebei region. Urban Dev Stud. (2017) 24:45–53.

[27] Pang S Dou S Li H. Synergy effect of science and technology policies on innovation: evidence from China. PLoS One. (2020) 15:e0240515. doi: 10.1371/journal.pone.0240515, 33048974 PMC7553322

[28] Hu X Yu S Fang X Ovaere M. Which combinations of renewable energy policies work better? Insights from policy text synergies in China. Energy Econ. (2023) 127:107104. doi: 10.1016/j.eneco.2023.107104

[29] Feng T Ma J Yang Y Mi Z. Synergistic effects of air pollution control policies: evidence from China. J Environ Manag. (2025) 373:123581. doi: 10.1016/j.jenvman.2024.123581, 39647296

[30] Zhu Y Wang K. Policy synergy analysis on coordinating reduction of pollution and carbon emissions in China: insights from text mining and network analysis. J Environ Manag. (2024) 372:123389. doi: 10.1016/j.jenvman.2024.123389, 39577188

[31] Wang Y Liu Y Du G Liu Y Zeng Y. Epidemiology and distribution of 207 rare diseases in China: a systematic literature review. Intractable Rare Dis Res. (2024) 13:73–88. doi: 10.5582/irdr.2024.01001, 38836174 PMC11145401

[32] Xinhua News Agency. (2024). Not ignoring small groups! China’s over 90 rare disease medicines into the medical insurance. Available online at: https://www.gov.cn/lianbo/bumen/202412/content_6991514.htm (Accessed March 29, 2025).

[33] Wang G. (2022). FAQ on rare disease drugs (orphan drugs) in China. Available online at: https://baipharm.chemlinked.com/insights/rare-disease-drug-orphan-drug-in-china (Accessed March 29, 2025).

[34] Yang Y Kang Q Hu J Kong F Tang M He J . Accessibility of drugs for rare diseases in China: policies and current situation. Intractable Rare Dis Res. (2019) 8:80–8. doi: 10.5582/irdr.2019.01068, 31218157 PMC6557233

[35] Ying Z Gong L Li C. An update on China’s national policies regarding rare diseases. Intractable Rare Dis Res. (2021) 10:148–53. doi: 10.5582/irdr.2021.01027, 34466336 PMC8397819

[36] Li X Wu L Yu L He Y Wang M Mu Y. Policy analysis in the field of rare diseases in China: a combined study of content analysis and bibliometrics analysis. Front Med (Lausanne). (2023) 10:1180550. doi: 10.3389/fmed.2023.1180550, 37215703 PMC10196157

[37] Wang X Huang L Daim T Li X Li Z. Evaluation of China’s new energy vehicle policy texts with quantitative and qualitative analysis. Technol Soc. (2021) 67:101770. doi: 10.1016/j.techsoc.2021.101770

[38] Liu D Wang D. Evaluation of the synergy degree of industrial de-capacity policies based on text mining: a case study of China’s coal industry. Resour Policy. (2022) 76:102547. doi: 10.1016/j.resourpol.2021.102547

[39] Dan H Yahua T. Evolution, coordination and textual content analysis of China’s green service policy. Chin J Environ Manage. (2021) 13:92–101. doi: 10.16868/j.cnki.1674-6252.2021.03.092

[40] Chen J Wang D Wang Q Cai Q. Quantifying the synergy of China’s carbon neutrality policies through policy documents. Renew Sust Energ Rev. (2024) 200:114585. doi: 10.1016/j.rser.2024.114585

[41] Yao C Sun M Liu L. Evaluation of policy synergy in coastal ocean pollution prevention and control: the case from China. Front Mar Sci. (2023) 10:1131590. doi: 10.3389/fmars.2023.1131590

[42] Zhu Y Hu Y Zhu Y. Can China’s energy policies achieve the “dual carbon” goal? A multi-dimensional analysis based on policy text tools. Environ Dev Sustain. (2024) 28:5551–90. doi: 10.1007/s10668-024-05190-4

[43] Zhang C Li X Sun Y Chen J Streimikiene D. Policy modeling consistency analysis during energy crises: evidence from China’s coal power policy. Technol Forecast Soc Change. (2023) 197:122931. doi: 10.1016/j.techfore.2023.122931

[44] Chong Z Wang Q Wang L. Is the photovoltaic power generation policy effective in China? A quantitative analysis of policy synergy based on text mining. Technol Forecast Soc Change. (2023) 195:122770. doi: 10.1016/j.techfore.2023.122770

[45] Yu J-K Li Y-H. Evolution of marine spatial planning policies for mariculture in China: overview, experience and prospects. Ocean Coast Manag. (2020) 196:105293. doi: 10.1016/j.ocecoaman.2020.105293

[46] Yu J Bi W. Evolution of marine environmental governance policy in China. Sustainability. (2019) 11:5076. doi: 10.3390/su11185076

[47] Scott J. Social network analysis. Sociology. (1988) 22:109–27. doi: 10.1177/0038038588022001007

[48] Borgatti SP. NetDraw: Graph Visualization Software. Harvard: Analytic Technologies (2002).

[49] Gao JJ Song PP Tang W. Rare disease patients in China anticipate the sunlight of legislation. Drug Discov Ther. (2013) 7:126–8. doi: 10.5582/ddt.2013.v7.3.126 23917862

[50] Gentilini A Neez E Wong-Rieger D. Rare disease policy in high-income countries: an overview of achievements, challenges, and solutions. Value Health. (2025) 28:680–5. doi: 10.1016/j.jval.2024.12.009, 39880194

[51] Wang L Zou H Ye F Wang K Li X Chen Z . Household financial burden of phenylketonuria and its impact on treatment in China: a cross-sectional study. J Inherit Metab Dis. (2017) 40:369–76. doi: 10.1007/s10545-016-9995-0, 27832415 PMC5393103

[52] Yi H Shi F Wang Z Kuai L Xu D Xie Y . Impacts of adjustment of national reimbursement drug list on orphan drugs volume and spending in China: an interrupted time series analysis. BMJ Open. (2023) 13:e064811. doi: 10.1136/bmjopen-2022-064811, 37852769 PMC10603398

[53] Wang X Li SC Yue X Li Y Shi N Zhao FL . Patient access to orphan drugs covered by medical insurance in China: real-world evidence from patient survey. Value Health Reg Issues. (2023) 34:71–7. doi: 10.1016/j.vhri.2022.10.008, 36587572

[54] Li L He X Li Y Chen X Wang X Li C. Understanding the impact of unequal utilization of medical services on the consumption of resources in hemophilia. Cost Eff Resour Alloc. (2022) 20:74. doi: 10.1186/s12962-022-00409-5, 36585723 PMC9805243

[55] Malinowski KP Kawalec P Trabka W Sowada C Pilc A. Reimbursement of orphan drugs in Europe in relation to the type of authorization by the European medicines agency and the decision making based on health technology assessment. Front Pharmacol. (2018) 9:1263. doi: 10.3389/fphar.2018.01263, 30483124 PMC6240661

[56] Sarnola K Ahonen R Martikainen JE Timonen J. Policies and availability of orphan medicines in outpatient care in 24 European countries. Eur J Clin Pharmacol. (2018) 74:895–902. doi: 10.1007/s00228-018-2457-x, 29632962

[57] Picavet E Annemans L Cleemput I Cassiman D Simoens S. Market uptake of orphan drugs—a European analysis. J Clin Pharm Ther. (2012) 37:664–7. doi: 10.1111/j.1365-2710.2012.01364.x, 22731105

[58] Zelei T Molnár MJ Szegedi M Kaló Z. Systematic review on the evaluation criteria of orphan medicines in central and eastern European countries. Orphanet J Rare Dis. (2016) 11:72. doi: 10.1186/s13023-016-0455-6, 27259284 PMC4893267

[59] Czech M Baran-Kooiker A Atikeler K Demirtshyan M Gaitova K Holownia-Voloskova M . A review of rare disease policies and orphan drug reimbursement systems in 12 Eurasian countries. Front Public Health. (2019) 7:416. doi: 10.3389/fpubh.2019.00416, 32117845 PMC6997877

[60] Fasseeh AN Korra N Aljedai A Seyam A Almudaiheem H Al-Abdulkarim HA . Rare disease challenges and potential actions in the Middle East. Int J Equity Health. (2025) 24:56. doi: 10.1186/s12939-025-02388-4, 40011905 PMC11863865

[61] Ministry of Health (2019). Rare disease fund to provide financial support to Singaporeans with rare diseases. Available online at: https://www.moh.gov.sg/newsroom/rare-disease-fund-to-provide-financial-support-to-singaporeans-with-rare-diseases (Accessed May 2, 2025).

