# Supplementary material for: Evolution and synergy of healthcare security policies for rare diseases in China: a quantitative content analysis
Source: Front Public Health. 2026 Jul 21;14:1864284. doi: 10.3389/fpubh.2026.1864284 (PMC13433357; doi:10.3389/fpubh.2026.1864284)
Supplement: Supplementary file 1 [file Table_1.pdf]

Supplementary Material

1 Government websites used for document search

Supplementary Table 1. Government websites used for document search

**Divisions/departments**	**Website**
Central People's Government of the People's Republic of China	https://www.gov.cn/
Ministry of Human Resources and Social Security of the People's Republic of China	https://www.mohrss.gov.cn/
National Healthcare Security Administration	https://www.nhsa.gov.cn/
National Health Commission of the People's Republic of China	http://www.nhc.gov.cn/
Ministry of Civil Affairs of the People's Republic of China	https://www.mca.gov.cn/
Tianjin Municipal People's Government	https://www.tj.gov.cn/
Tianjin Municipal Human Resources and Social Security Bureau	https://hrss.tj.gov.cn/
Tianjin Municipal Healthcare Security Administration	https://ylbz.tj.gov.cn/
Tianjin Municipal Health Commission	https://wsjk.tj.gov.cn/
Tianjin Municipal Civil Affairs Bureau	https://mz.tj.gov.cn/
Chongqing Municipal People's Government	https://www.cq.gov.cn/
Chongqing Municipal Human Resources and Social Security Bureau	https://rlsbj.cq.gov.cn/
Chongqing Municipal Healthcare Security Administration	https://ylbzj.cq.gov.cn/
Chongqing Municipal Health Commission	https://wsjkw.cq.gov.cn/
Chongqing Municipal Civil Affairs Bureau	https://mzj.cq.gov.cn/
Henan Province People's Government	https://www.henan.gov.cn/
Henan Province Human Resources and Social Security Department	https://hrss.henan.gov.cn/
Henan Province Healthcare Security Administration	https://ylbz.henan.gov.cn/
Henan Province Health Commission	https://wsjkw.henan.gov.cn/

Henan Province Civil Affairs Department	https://mzt.henan.gov.cn/
Hebei Province People's Government	https://www.hebei.gov.cn/
Hebei Province Human Resources and Social Security Department	https://rst.hebei.gov.cn/
Hebei Province Healthcare Security Administration	https://ylbzj.hebei.gov.cn/
Hebei Province Health Commission	http://wsjkw.hebei.gov.cn/
Hebei Province Civil Affairs Department	https://minzheng.hebei.gov.cn/
Shaanxi Provincial People's Government	https://www.shaanxi.gov.cn/
Shaanxi Province Human Resources and Social Security Department	https://rst.shaanxi.gov.cn/
Shaanxi Province Healthcare Security Administration	https://ybj.shaanxi.gov.cn/
Shaanxi Province Health Commission	https://sxwjw.shaanxi.gov.cn/
Shaanxi Province Civil Affairs Department	https://mzt.shaanxi.gov.cn/
Yunnan Province People's Government	https://www.yn.gov.cn/
Yunnan Province Human Resources and Social Security Department	https://hrss.yn.gov.cn/
Yunnan Province Healthcare Security Administration	https://ylbz.yn.gov.cn/
Yunnan Province Health Commission	https://ynswsjkw.yn.gov.cn/
Yunnan Province Civil Affairs Department	https://ynmz.yn.gov.cn/
Heilongjiang Province People's Government	https://www.hlj.gov.cn/
Heilongjiang Province Human Resources and Social Security Department	https://hrss.hlj.gov.cn/
Heilongjiang Province Healthcare Security Administration	https://ybj.hlj.gov.cn/
Heilongjiang Province Health Commission	http://wsjkw.hlj.gov.cn/
Heilongjiang Province Civil Affairs Department	https://mzt.hlj.gov.cn/
Jiangxi Province People's Government	https://www.jiangxi.gov.cn/
Jiangxi Province Human Resources and Social Security Department	https://rst.jiangxi.gov.cn/
Jiangxi Province Healthcare Security Administration	https://ybj.jiangxi.gov.cn/
Jiangxi Province Health Commission	https://hc.jiangxi.gov.cn/
Jiangxi Province Civil Affairs Department	https://mzt.jiangxi.gov.cn/
Shanghai Municipal People's Government	https://www.shanghai.gov.cn/

Shanghai Municipal Human Resources and Social Security Bureau	https://rsj.sh.gov.cn/
Shanghai Municipal Healthcare Security Administration	https://ybj.sh.gov.cn/
Shanghai Municipal Health Commission	https://wsjkw.sh.gov.cn/
Shanghai Municipal Civil Affairs Bureau	https://mzj.sh.gov.cn/
Zhejiang Province People’s Government	https://www.zj.gov.cn/
Zhejiang Province Human Resources and Social Security Department	https://rlsbt.zj.gov.cn/
Zhejiang Province Healthcare Security Administration	http://ybj.zj.gov.cn/
Zhejiang Province Health Commission	https://wsjkw.zj.gov.cn/
Zhejiang Province Civil Affairs Department	https://mzt.zj.gov.cn/
Anhui Province People’s Government	https://www.ah.gov.cn/
Anhui Province Human Resources and Social Security Department	https://hrss.ah.gov.cn/
Anhui Province Healthcare Security Administration	https://ybj.ah.gov.cn/
Anhui Province Health Commission	https://wjw.ah.gov.cn/
Anhui Province Civil Affairs Department	http://mz.ah.gov.cn/
Jilin Province People’s Government	https://www.jl.gov.cn/
Jilin Province Human Resources and Social Security Department	http://hrss.jl.gov.cn/
Jilin Province Healthcare Security Administration	http://ybj.jl.gov.cn/
Jilin Province Health Commission	http://wsjkw.jl.gov.cn/
Jilin Province Civil Affairs Department	http://mzt.jl.gov.cn/
Beijing Municipal People’s Government	https://www.beijing.gov.cn/
Beijing Municipal Human Resources and Social Security Bureau	https://rsj.beijing.gov.cn/
Beijing Municipal Healthcare Security Administration	https://ybj.beijing.gov.cn/
Beijing Municipal Health Commission	https://wjw.beijing.gov.cn/
Beijing Municipal Civil Affairs Bureau	https://mzj.beijing.gov.cn/
Liaoning Province People’s Government	https://www.ln.gov.cn/
Liaoning Province Human Resources and Social Security Department	https://rst.ln.gov.cn/
Liaoning Province Healthcare Security Administration	https://ybj.ln.gov.cn/

Liaoning Province Health Commission	https://wsjk.ln.gov.cn/
Liaoning Province Civil Affairs Department	https://mzt.ln.gov.cn/
Qinghai Province People's Government	http://www.qinghai.gov.cn/
Qinghai Province Human Resources and Social Security Department	https://rst.qinghai.gov.cn/
Qinghai Province Healthcare Security Administration	https://ybj.qinghai.gov.cn/
Qinghai Province Health Commission	https://wsjkw.qinghai.gov.cn/
Qinghai Province Civil Affairs Department	https://mzt.qinghai.gov.cn/
Shanxi Province People's Government	https://www.shanxi.gov.cn/
Shanxi Province Human Resources and Social Security Department	https://rst.shanxi.gov.cn/
Shanxi Province Healthcare Security Administration	https://ybj.shanxi.gov.cn/
Shanxi Province Health Commission	https://wjw.shanxi.gov.cn/
Shanxi Province Civil Affairs Department	https://mzt.shanxi.gov.cn/
Shandong Province People's Government	https://www.shandong.gov.cn/
Shandong Province Human Resources and Social Security Department	http://hrss.shandong.gov.cn/
Shandong Province Healthcare Security Administration	http://ybj.shandong.gov.cn/
Shandong Province Health Commission	http://wsjkw.shandong.gov.cn/
Shandong Province Civil Affairs Department	http://mzt.shandong.gov.cn/
Xinjiang Uyghur Autonomous Region People's Government	https://www.xinjiang.gov.cn/
Xinjiang Uyghur Autonomous Region Human Resources and Social Security Department	https://rst.xinjiang.gov.cn/
Xinjiang Uyghur Autonomous Region Healthcare Security Administration	https://ylbzj.xinjiang.gov.cn/
Xinjiang Uyghur Autonomous Region Health Commission	https://wjw.xinjiang.gov.cn/
Xinjiang Uyghur Autonomous Region Civil Affairs Department	https://mzt.xinjiang.gov.cn/
Xinjiang Production and Construction Corps People's Government	http://www.xjbt.gov.cn/
Xinjiang Production and Construction Corps Human Resources and Social Security Bureau	http://rsj.xjbt.gov.cn/
Xinjiang Production and Construction Corps Healthcare Security Administration	http://btybj.xjbt.gov.cn/
Xinjiang Production and Construction Corps Health Commission	http://wsj.xjbt.gov.cn/
Xinjiang Production and Construction Corps Civil Affairs Bureau	http://mzj.xjbt.gov.cn/

Fujian Province People's Government	https://www.fujian.gov.cn/
Fujian Province Human Resources and Social Security Department	https://rst.fujian.gov.cn/
Fujian Province Healthcare Security Administration	https://ybj.fujian.gov.cn/
Fujian Province Health Commission	https://wjw.fujian.gov.cn/
Fujian Province Civil Affairs Department	https://mzt.fujian.gov.cn/
Hubei Province People's Government	https://www.hubei.gov.cn/
Hubei Province Human Resources and Social Security Department	https://rst.hubei.gov.cn/
Hubei Province Healthcare Security Administration	https://ybj.hubei.gov.cn/
Hubei Province Health Commission	https://wjw.hubei.gov.cn/
Hubei Province Civil Affairs Department	https://mzt.hubei.gov.cn/
Ningxia Hui Autonomous Region People's Government	https://www.nx.gov.cn/
Ningxia Hui Autonomous Region Human Resources and Social Security Department	https://hrss.nx.gov.cn/
Ningxia Hui Autonomous Region Healthcare Security Administration	https://ylbz.nx.gov.cn/
Ningxia Hui Autonomous Region Health Commission	https://wsjkw.nx.gov.cn/
Ningxia Hui Autonomous Region Civil Affairs Department	http://mca.nx.gov.cn/
Hunan Province People's Government	https://www.hunan.gov.cn/
Hunan Province Human Resources and Social Security Department	https://rst.hunan.gov.cn/
Hunan Province Healthcare Security Administration	https://ybj.hunan.gov.cn/
Hunan Province Health Commission	https://wjw.hunan.gov.cn/
Hunan Province Civil Affairs Department	https://mzt.hunan.gov.cn/
Gansu Province People's Government	https://www.gansu.gov.cn/
Gansu Province Human Resources and Social Security Department	https://rst.gansu.gov.cn/
Gansu Province Healthcare Security Administration	https://ylbz.gansu.gov.cn/
Gansu Province Health Commission	https://wsjk.gansu.gov.cn/
Gansu Province Civil Affairs Department	https://mzt.gansu.gov.cn/
Guizhou Province People's Government	https://www.guizhou.gov.cn/
Guizhou Province Human Resources and Social Security Department	https://rst.guizhou.gov.cn/

Guizhou Province Healthcare Security Administration	https://ylbzj.guizhou.gov.cn/
Guizhou Province Health Commission	https://wjw.guizhou.gov.cn/
Guizhou Province Civil Affairs Department	https://mzt.guizhou.gov.cn/
Jiangsu Province People's Government	https://www.jiangsu.gov.cn/
Jiangsu Province Human Resources and Social Security Department	https://jshrss.jiangsu.gov.cn/
Jiangsu Province Healthcare Security Administration	https://ybj.jiangsu.gov.cn/
Jiangsu Province Health Commission	https://wjw.jiangsu.gov.cn/
Jiangsu Province Civil Affairs Department	https://mzt.jiangsu.gov.cn/
Sichuan Province People's Government	https://www.sc.gov.cn/
Sichuan Province Human Resources and Social Security Department	https://rst.sc.gov.cn/
Sichuan Province Healthcare Security Administration	https://ylbzj.sc.gov.cn/
Sichuan Province Health Commission	https://wsjkw.sc.gov.cn/
Sichuan Province Civil Affairs Department	https://mzt.sc.gov.cn/
Inner Mongolia Autonomous Region People's Government	https://www.nmg.gov.cn/
Inner Mongolia Autonomous Region Human Resources and Social Security Department	https://rst.nmg.gov.cn/
Inner Mongolia Autonomous Region Healthcare Security Administration	https://ylbzj.nmg.gov.cn/
Inner Mongolia Autonomous Region Health Commission	https://wjw.nmg.gov.cn/
Inner Mongolia Autonomous Region Civil Affairs Department	https://mzt.nmg.gov.cn/
Hainan Province People's Government	https://www.hainan.gov.cn/
Hainan Province Human Resources and Social Security Department	https://hrss.hainan.gov.cn/
Hainan Province Healthcare Security Administration	https://ybj.hainan.gov.cn/
Hainan Province Health Commission	https://wst.hainan.gov.cn/
Hainan Province Civil Affairs Department	https://mz.hainan.gov.cn/
Guangdong Province People's Government	https://www.gd.gov.cn/
Guangdong Province Human Resources and Social Security Department	https://hrss.gd.gov.cn/
Guangdong Province Healthcare Security Administration	https://hsa.gd.gov.cn/
Guangdong Province Health Commission	https://wsjkw.gd.gov.cn/

Guangdong Province Civil Affairs Department	https://smzt.gd.gov.cn/
Tibet Autonomous Region People's Government	https://www.xizang.gov.cn/
Tibet Autonomous Region Human Resources and Social Security Department	https://hrss.xizang.gov.cn/
Tibet Autonomous Region Healthcare Security Administration	https://ylbzj.xizang.gov.cn/
Tibet Autonomous Region Health Commission	https://wjw.xizang.gov.cn/
Tibet Autonomous Region Civil Affairs Department	https://mzt.xizang.gov.cn/
Guangxi Zhuang Autonomous Region People's Government	http://www.gxzf.gov.cn/
Guangxi Zhuang Autonomous Region Human Resources and Social Security Department	http://rst.gxzf.gov.cn/
Guangxi Zhuang Autonomous Region Healthcare Security Administration	http://ybj.gxzf.gov.cn/
Guangxi Zhuang Autonomous Region Health Commission	https://wsjkw.gxzf.gov.cn/
Guangxi Zhuang Autonomous Region Civil Affairs Department	http://mzt.gxzf.gov.cn/
